# Immunodiagnostic usefulness of monoclonal antibodies specific to conformational epitopes of *Taenia solium* oncosphere protein TSOL18

**DOI:** 10.1016/j.jim.2021.113121

**Published:** 2021-10

**Authors:** Emmanuel Assana, André Pagnah Zoli, Charles G. Gauci, Marshall W. Lightowlers, Pierre Dorny

**Affiliations:** a University of Ngaoundéré, School of Veterinary Medicine and Sciences, P.O. Box 454, Ngaoundéré, Cameroon; b The University of Melbourne, Faculty of Veterinary and Agricultural Sciences, Veterinary Clinical Centre, 250 Princes Highway, Werribee, Victoria 3030, Australia; c Institute of tropical Medicine, Department of Biomedical Sciences, Nationale Straat 155, B-2000 Antwerp, Belgium

**Keywords:** *Taenia solium*, TSOL18, Monoclonal antibody, Epitopes, Immunodiagnosis

## Abstract

*Taenia solium* oncosphere protein TSOL18 is the host-protective antigen against porcine cysticercosis. Little attention has been given to use it as target molecule in immunodiagnostic tests. The objective of this paper is to describe the immunodiagnostic potential of monoclonal antibodies (MoAbs) raised against conformational epitopes of TSOL18. Three murine IgG1 MoAbs (25D12C1, 21C2D2, 10H1F2) against three different conformational epitopes of TSOL18 were produced and evaluated with an inhibition enzyme-linked immunosorbent assay (i-ELISA) for the detection of anti-TSOL18 and anti-oncosphere antibodies. Serum samples from pigs immunized with TSOL18 inhibited the binding of the three MoAbs to TSOL18 antigen in i-ELISA. The highest inhibition of anti-TSOL18 antibodies in immunized pigs was observed with MoAb 25D12C1. Ten field sera (12.19%) from 82 non-vaccinated and non-infected pigs showed anti-oncosphere antibodies inhibiting the binding of MoAb 25D12C1. Anti-oncosphere antibodies in pigs experimentally infected with *T. solium* eggs inhibited the binding of MoAb 25D12C1 from 2 to 8 week-post infection. It is concluded that MoAb 25D12C1 has excellent immunodiagnostic potentials to detect anti-oncosphere antibodies in the intermediate hosts at early exposure to *T. solium* eggs. Further investigations on potential use of MoAb 25D12C1 in a capture antigen ELISA for the detection of post-oncospheral antigens in infected pigs cannot be overemphasized.

## Highlights

• We describe the potential usefulness of the monoclonal antibody 25D12C1 to TSOL18 protein.
• Serum samples from pigs immunized with TSOL18 inhibited the binding of 25D12C1 in an inhibition enzyme-linked immunosorbent assay (i-ELISA) for the detection of anti-TSOL18 and anti-oncosphere antibodies.
• Anti-oncosphere antibodies in pigs infected with *T. solium* eggs inhibited also the binding of 25D12C1.
• It is concluded that MoAb i-ELISA using 25D12C1 has the potential to identify animals exposed to *T. solium* infection.

## Graphical abstract

**Figure:**
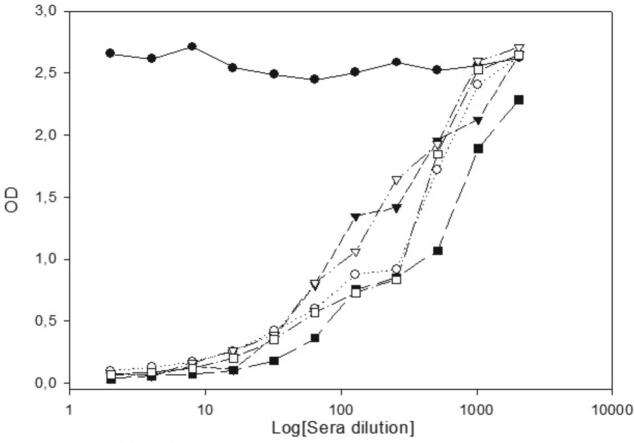

## 1 Introduction

*Taenia solium* taeniasis/cysticercosis has a complex two-host life cycle with pigs as intermediate hosts harboring the larval stages or metacestodes and humans as both aberrant intermediate hosts and definitive hosts harboring the adult tapeworm in the small intestine. The disease affects pig production and has considerable public health implications as a zoonosis in low and middle income countries (Gabriël et al., 2017; Dixon et al., 2019). The species-specific diagnosis of *T. solium* cysticercosis in pigs is important for effective assessment of the control measures against the *T. solium* taeniasis and cysticercosis in humans. Currently, the definite method for diagnosis of porcine cysticercosis is the detection of cysticerci at necropsy (Dorny et al., 2004; Lightowlers et al., 2016; Lightowlers, 2020). Attempts have been made to develop species-specific immunodiagnostic tool for diagnosis of the parasite in infected pigs using different antigens from *T. solium* (Ito and Craig, 2003). However, little attention has been given to the immunodiagnostic usefulness of TSOL18 protein, a recombinant oncosphere antigen of *T. solium*. The antigen is expressed at the surface of hatched oncospheres and not in the adult tapeworm or in fully developed metacestodes, indicating that it is a stage-specific antigen (Gauci et al., 2006; Kyngdon et al., 2006; Matinez-Ocana et al., 2011). This suggests that postoncospheral antigens excreted at early exposure of pigs to *T. solium* eggs can be detected using anti-TSOL18 antibodies. Further investigations have shown that antibody responses to TSOL18 are directed against conformational epitopes (Assana et al., 2010a). Therefore, Immunodiagnostic tool using monoclonal antibodies against conformational epitopes of TSOL18 to detect anti- oncospheral antibodies or oncospheral antigen and not cysticercal antigens would be of interest in epidemiological studies, particularly for the detection of pigs (and also humans) with exposure to *T. solium* eggs. In this context, this study was carried out to evaluate the efficiency of murine MoAbs against conformational epitopes of TSOL18 to inhibit the binding of anti-oncosphere antibodies to TSOL18 in i-ELISA using serum sample from pigs infected with *T. solium* eggs.

## 2 Materials and methods

### 2.1 Antigens

*T. solium* oncosphere recombinant antigen fused to glutathione S-transferase (TSOL18-GST) was prepared as previously described (Flisser et al., 2004). This antigen was also expressed as a maltose binding protein (MBP) fusion (Maïna et al., 1988). Soluble TSOL18-GST and TSOL18-MBP were affinity purified from *E. coli* proteins using glutathione-Sepharose (Amersham bioscience Uppsala, Sweden) and maltose beads (Biolabs, New England, UK), respectively. In addition, two truncated TSOL18 proteins (TSOL18-1, TSOL18-2) were expressed in *E. coli* as GST fusion proteins. While TSOL18-1 and TSOL18-2 were produced by cloning PCR-amplified truncated TSOL18 cDNA into the pGEX-1 TEX vector. TSOL18-1 contained 71 amino acids starting from the amino terminus of TSOL18. TSOL18-2 consisted of the last 66 amino acids of TSOL18. These two truncated proteins overlapped each other by 25 amino acids. All the steps of production and purification of truncated TSOL18 proteins fused with glutathione S-transferase are identical for the whole protein TSOL18-GST.

### 2.2 Production of monoclonal antibodies (MoAbs) to TSOL18 antigen

BALB/c female mice were immunized subcutaneously, twice at two weeks intervals, with 10 μg of TSOL18-MBP and 5 μg of Quil A adjuvant (Superfos Biosector, Vedbaek, Denmark). A boost of TSOL18-GST was given intramuscularly three days before the fusion. Spleen cells from the immunized mouse were fused with myeloma cells (NSO). Ten days after the fusion, supernatants of hybridoma were screened using an indirect ELISA. Positive cells were cloned two times by limiting dilution. The clones were screened and positive clones selected for MoAb production and grown in a cell culture (RPMI-1640 with 10% FCS, Gibco). i-ELISA previously described by Assana et al. (2010a) was used for the selection of MoAbs against the conformational epitopes of TSOL18. The MoAbs isotypes were determined by an immunoassay kit (Southern Biotechnology, Associates, USA) using MoAbs against mouse immunoglobulins. The produced MoAbs were purified and labeled using a biotin protein labeling kit (Roche Diagnostics, Mannheim, Germany).

### 2.3 Monoclonal antibody-based inhibition enzyme-linked immunosorbent assay (i-ELISA) specific to conformational epitopes of TSOL18 vaccine

#### 2.3.1 Serum samples

Serum samples from Australian pigs (*n* = 5) experimentally immunized with TSOL18-GST and bled at two weeks post 2nd immunization were used as positive control serum samples in i-ELISAs in the present study. While serum samples collected prior to experimental immunization were used as negative control sera.

Also, two Australian pigs immunized with the two fragments of TSOL18 antigen (TSOL18-1 and TSOL18-2) were used as hyper-immune sera against the linear epitopes of TSOL18 protein.

A total of 212 sera from TSOL18-GST vaccinated (*n* = 110) and non-vaccinated (*n* = 102) Cameroonian pigs collected at necropsy (Assana et al., 2010b) were used as field sera.

Sera of 4 Cameroonian pigs, experimentally infected with different doses of *T. solium* eggs were obtained from the Institute of Tropical Medicine Antwerp (Belgium). The experimental infections were done with *T. solium* proglottids (2 pigs), which resulted in massive infections of more than 3000 cysticerci per kg of carcass, and with 10,000 *T. solium* eggs (2 pigs), which resulted in light infections of 19 and 14 cysticerci per carcass, respectively (Nguekam et al., 2003). Naïve sera collected prior to the immunization or experimental challenge infection were used as negative control sera in performed i-ELISA.

In addition, five serum samples from Australian pigs experimentally infected with *T. hydatigena* (*n* = 5) obtained from the University of Melbourne (Australia) were also used for analysis of cross- reactivity with *T. solium.*

#### 2.3.2 Monoclonal antibody-based inhibition ELISA procedure

A MoAb-based i-ELISA specific to the TSOL18 vaccine conformational epitopes was performed to determine: (1) competition between the various produced MoAbs and (2) competition between pig antibodies and MoAbs for the same conformational epitope on TSOL18. Briefly, Polysorb plates (Nunc®) were coated with TSOL18-MBP in carbonate bicarbonate buffer, pH 9.6. The plates were incubated with PBS-NBCS-TW20 at 37 °C. The test sera from pigs or MoAbs (0.02 mg/ml) were serially diluted in 11 different concentrations in PBS-NBCS-TW20 from 1/1 to 1/1024 and distributed on the plates and incubated for 1 h at 37 °C. The serum samples or MoAbs were washed from the plates and incubated for 40 min at 37 °C with biotinylated MoAbs. Then, the plates were incubated with streptavidine peroxidase-conjugate (Sigma) for 30 min at 37 °C and adding of chromogen/substrate solution consisting of orthophenylene diamine and H_2_O_2._ The color reaction was stopped by using 50 μ/well of 4 N H_2_SO_4_. The absorbance values were measured at 492 nm in an ELISA reader (Multiscan EX, Termo Labsystems). MoAb 6C6 to *Trichinella spiralis* obtained from the Institute of Tropical Medicine Antwerp (Belgium) was used as negative control in both biotinylated and non-biotinylated forms in the i-ELISA plates. The optimal positive inhibition was interpreted as the serum dilution at which optical densities (OD) decreased to levels approaching 0.

## 3 Results and discussion

Eleven MoAbs were raised to TSOL18 antigen (Table 1). Four MoAbs (IgG 1) specific to conformational epitopes designated 4A9B6, 10H1F2, 25D12C1 and 21C2D2 were selected for i-ELISA evaluation. TSOL18 inhibited the binding of the MoAbs to TSOL18 on the plate in i-ELISA. However, linear antigens (TSOL18-1 and TSOL18-2) caused no inhibition in i-ELISA (Table 1), suggesting that the four MoAbs recognized only TSOL18 vaccine conformational epitopes as previously demonstrated (Assana et al., 2010a).

**Table 1:** Characterization of anti-TSOL18 monoclonal antibodies (MoAbs).

Clone	MoAbs isotypes reacting in immunoassay kit (Southern Biotechnology, Associates, USA)						Reactivity of MoAbs with full length and the two truncated TSOL18 proteins in i-ELISA		
	IgM	IgG1	IgG2a	IgG2b	IgG3	IgA	TSOL18	TSOL18-1	TSOL18-2
4A9B6	−	+	−	−	−	−	+	−	−
21C2D2	−	+	−	−	−	−	+	−	−
25D12C1	−	+	−	−	−	−	+	−	−
10H1F2	−	+	−	−	−	−	+	−	−
19A2	+	−	−	−	−	−	+	−	−
22A12	−	+	−	−	−	−	+	−	−
2F4	−	+	−	−	−	−	+	−	−
15F1	+	−	−	−	−	−	+	−	+
13H8	+	−	−	−	−	−	+	−	+
21H7	+	−	−	−	−	−	+	−	−
25H7	−	+	−	−	−	−	+	−	−

+: Positive result in characterization of monoclonal antibodies.

-: Negative result in characterization of monoclonal antibodies.

**Fig. 1:**
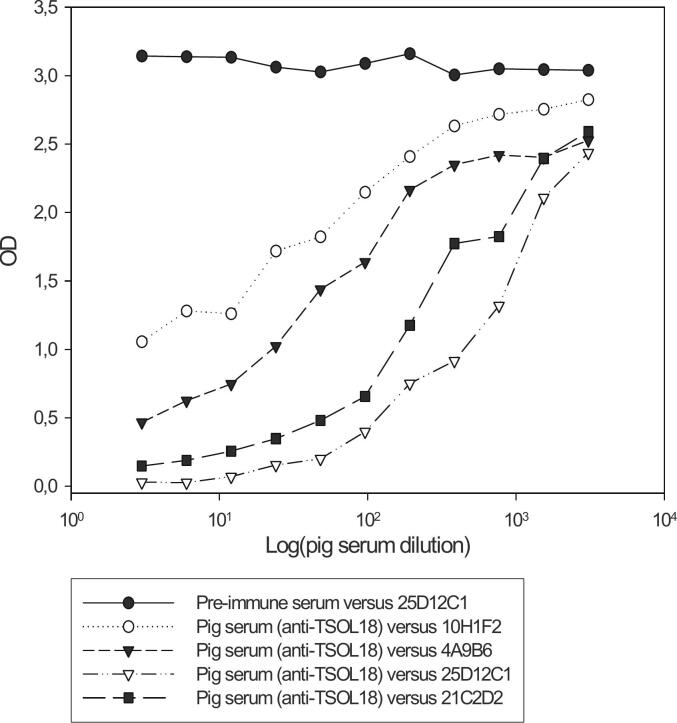
Competition between MoAbs and pig antisera to TSOL18. A serum from a pig experimentally immunized with TSOL18 and bled at two weeks post 2nd immunization was used in i-ELISA to bind to TSOL18 coated onto the ELISA plate. A pre-immune serum was used as negative control serum. Biotinylated MoAbs were added to the ELISA for competition with pig antiserum. The results indicated that pig antiserum inhibited the binding of all four MoAbs (4A9B6, 10H1F2, 25D12C1 and 21C2D2). The highest inhibition was observed with 25D12C1 MoAbs (OD decreased at the level approaching 0).

**Fig. 2:**
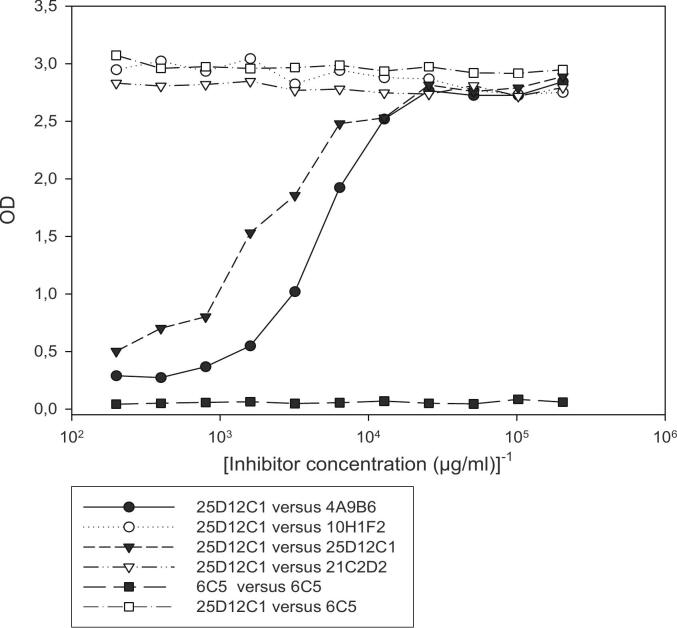
Competition between the 4A9B6, 10H1F2, 25D12C1 and 21C2D2 MoAbs. 4A9B6,10H1F2, 25D12C2 and 21C2D2 MoAbs were serially diluted and distributed on the plates previously coated with TSOL18 antigen. 25D12C1 biotinylated MoAb was used for analysing the inhibition. Biotinylated and non-biotinylated MoAb 6C5 against *Trichinella spiralis* antigen were used as negative MoAb controls. The results indicate that 25D12C1 and 4A9B6 MoAbs inhibited each other for the binding to TSOL18 antigen, suggesting that these two MoAbs are identical or specific for the same TSOL18epitope.

**Fig. 3:**
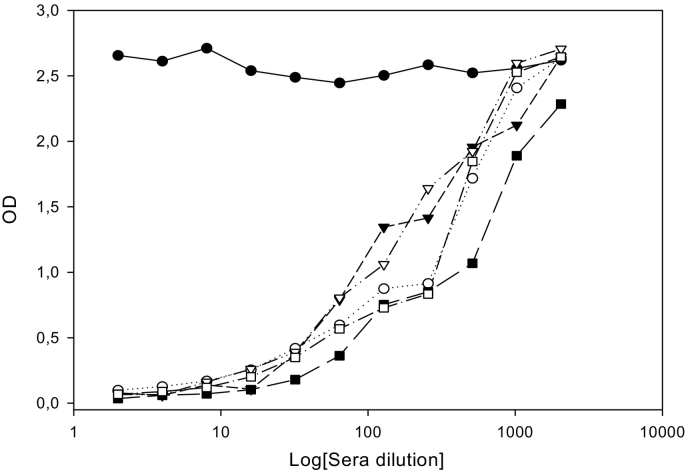
Optimization of i-ELISA using MoAb 25D12C1. Sera from 5 pigs experimentally immunized with TSOL18 (○▼∇ ￭ ⎕) bled at two week post 2nd immunization were used for the optimization of i-ELISA using MoAb 25D12C1. A pre-immune serum (●) was used as negative control serum. The optimal positive inhibition was interpreted as the serum dilution at which optical densities (OD) decreased to levels approaching 0.

Pig TSOL18 anti-sera inhibited the binding of all four MoAbs (Fig. 1). The competition between the MoAbs was analyzed as follows: 4A9B6 versus 10H1F2, 4A9B6 versus 25D12C1, 4A9B6 versus 21C2D2, 10H1F2 versus 25D12C1, 10H1F2 versus 21C2D2 and 25D12C1 versus 21C2D2 (Fig. 2). The 25D12C1 and 4A9B6 MoAbs inhibited each other from binding to TSOL18 antigen, suggesting that the two MoAbs were identical and recognized the same TSOL18 epitope. The other 25D12C1/4A9B6, 10H1F2 and 21C2D2 MoAbs did not inhibit each other, suggesting that 3 different epitopes were targeted by the MoAbs, confirming that the TSOL18 antigen is multiepitope. The optimal inhibition of the binding of pig antibody was observed with Mab MoAb 25DD12C1 (optical density decreased to almost zero). The optimal dilution of the biotin-conjugated 25D12C1 MoAbs (1/10000) was selected on the basis of a checkerboard titration that differentiated negative from positive sera of pigs immunized with TSOL18 (Fig. 3).

**Fig. 4:**
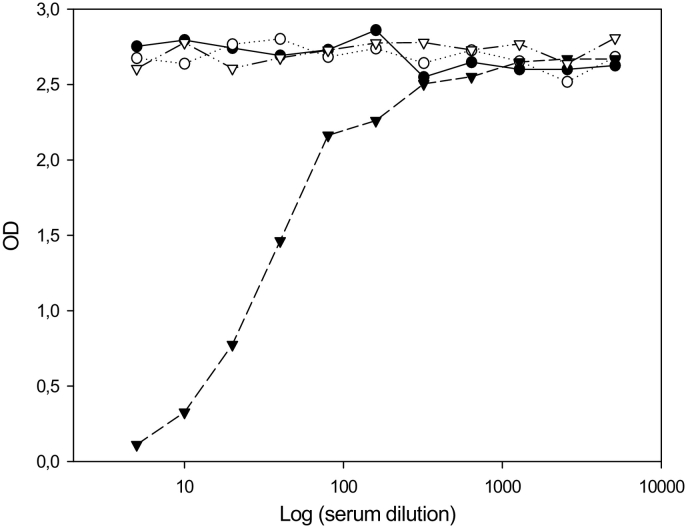
Results of i-ELISA using sera from pigs experimentally infected with *T. solium.* Two pigs (▼ and ●) were infected with mature proglottids and each of them developed more than 3000 cysticerci in the muscles. Two other pigs (∇ and ○) received 10,000 *T. solium* eggs and developed 19 and 14 cysticerci in the carcass, respectively (Nguekam et al., 2003). The sera were collected at four weeks post infection. One serum from a pig that received entire proglottids exhibited high inhibition of the binding of MoAb 25D12C (▼). The sera of this pig were used for screening the kinetics of anti-antibodies inhibiting the binding of 25D12C1 MoAbs from week 2 to week 10 post-infection (Fig. 5).

**Fig. 5:**
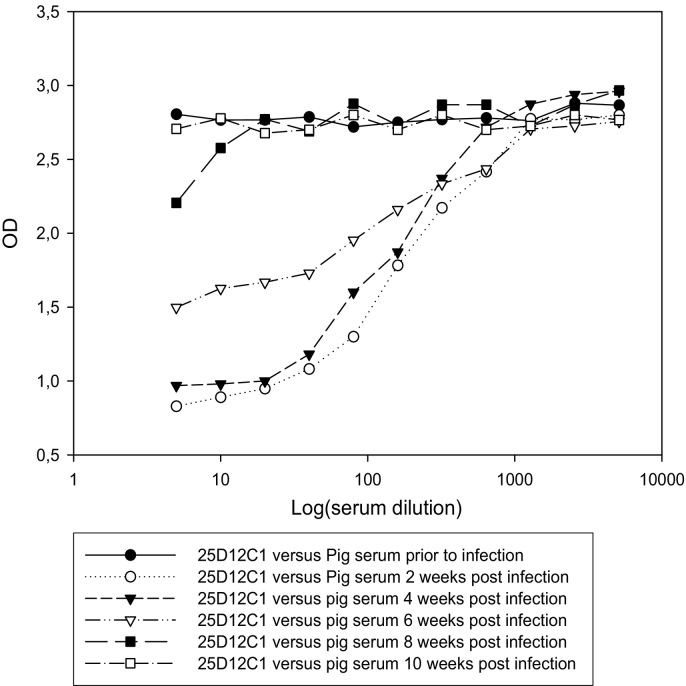
The kinetics of pig antibodies inhibiting the binding of MoAb 25D12C1 in one of the experimentally infected pigs that exhibited high level of inhibition of theMoAb. Anti-oncosphere antibodies inhibited the binding of MoAb 25D12C1 to TSOL18 antigen from 2 to 4 week-post infection. Ten weeks after the infection, the pig serum was found negative, indicating a low level or a disappearance of anti- oncosphere antibodies inhibiting the binding of MoAb 25D12C1(⎕).

The i-ELISA results using sera from pigs experimentally infected with *T. solium* eggs are shown in Fig. 4. One serum from a pig that received entire proglottids of *T. solium* exhibited strong inhibition of the binding of MoAb 25D12C1. The kinetics of anti-oncosphere antibodies inhibiting the binding of MoAb 25D12C1 in sera of the infected pig with entire proglottids were screened. Ten weeks post-infection, anti-oncosphere IgG1 antibodies that inhibited the binding of MoAb 25D12C1 were not detectable anymore in the serum, suggesting a decrease and or disappearance of the antibodies to post-oncospheral antigens (Fig. 5). This is in agreement with previous observations that the TSOL18 antigen is secreted by the hatched oncosphere and the early stage of the development of cysticerci and not by mature cysticerci (Gauci et al., 2006; Kyngdon et al., 2006; Matinez-Ocana et al., 2011). In the present study, serum of 10 (12.19%) of 82 pigs found negative at necropsy inhibited the binding of MoAb 25D12C1 to TSOL18, suggesting early infection with native oncospheres (Table 2). There is clear evidence that transient serological responses occur in pigs, including in Ab-ELISA, following exposure to *T. solium* eggs even if the parasites do not complete maturation (Devleesschauwer et al., 2013; Garcia et al., 2001). In addition, pigs that undergo comprehensive necropsy to diagnose infection may fail to detect early developing cysticerci before they reach a size that can be identified macroscopically (Chembensofu et al., 2017). The findings described here suggest that 25D12C1 MoAb i-ELISA can be useful for the detection of anti-oncospheral antibodies at two weeks post-infection, at which time metacestodes are small and not visible during carcass inspection.

**Table 2:** Results of i-ELISA using field sera* from vaccinated and non-vaccinated pigs bled at necropsy.

Pig status	Necropsy	MoAb-i-ELISA	Number of pigs
Vaccinated	Non-infected	+	110
Vaccinated	Non-infected	−	0
Non-vaccinated	Infected	+	7
Non-vaccinated	Infected	−	13
Non-vaccinated	Non-infected	+	10
Non-vaccinated	Non-infected	−	72

*Sera were collected at necropsy (24 week post 3rd immunization boost). In a previous study, none of the vaccinated pigs (110) were found infected whereas 20 of 102 non-vaccinated pigs were infected with cysticerci (Assana et al., 2010a).

+ = Positive (pig antibodies inhibiting the binding of 25D12C1 MoAb to TSOL18).

- = Negative (no inhibition of the binding of 25D12C1 MoAb to TSOL18).

The current monoclonal antibody-based Ag-ELISA detects circulating excretory-secretory antigens of cysticerci and not oncospheral antigens (Brandt et al., 1992; Paredes et al., 2016; Bustos et al., 2019). Obviously, in epidemiological studies, these tests cannot measure proportion of pigs and humans with exposure to *T. solium* eggs that does not lead to the establishment of cysticerci. MoAb i-ELISA using 25D12C1 has the potential to identify animals recently exposed to *T. solium* infection.

**Fig. 6:**
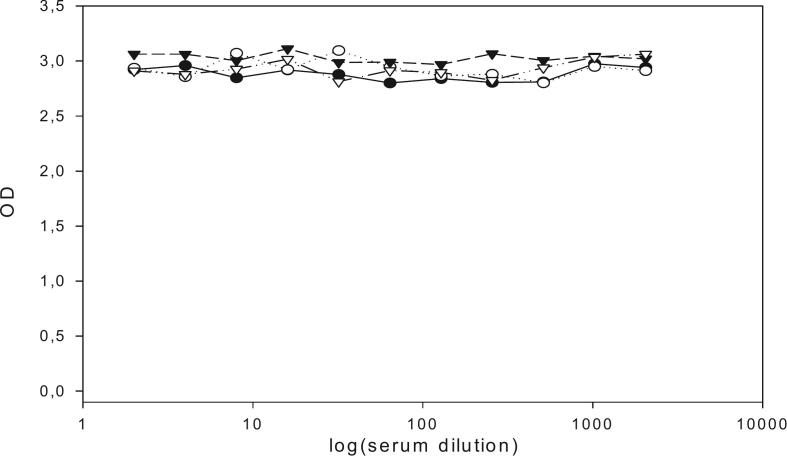
Result of i-ELISA using serum samples of pigs experimentally infected with *T. hydatigena*. In the same way as shown in Fig. 4, sera of pigs experimentally infected with *T. hydatigena* were used in i-ELISA. These sera caused no inhibition of the binding site of MoAb 25D12C1 on TSOL18, indicating no cross-reactivity between antibody to *T. hydatigena* and MoAb 25D12C1.

MoAb 25D12C1 do not show cross-reaction with serum samples of pigs experimentally infected with *T. hydatigena* (Fig. 6). *T. solium, T. hydatigena* and *Taenia asiatica* are all taeniid cestodes that infect pigs. Although potential cross-reactivity between MoAb 25D12C1 and antibodies in serum raised by exposure to *T. asiatica* was not investigated here, it is *T. hydatigena* which has the widest global distribution and presents the most concern about serological cross-reactivity in assays for *T. solium* cysticercosis in pigs (Lightowlers et al., 2016). From the evidence presented in Fig. 6 it appears that antibodies that cross-react with MoAb 25D12C1 are not raised in pigs by exposure to *T. hydatigena* and hence MoAb i-ELISA using 25D12C1 has appropriate specificity to accurately diagnose early infections with *T. solium* in pigs.

## Declaration of competing interest

None.
